# The prognostic value of IPI in patients with primary breast lymphoma, a multicenter retrospective study

**DOI:** 10.1186/s12935-022-02772-y

**Published:** 2022-11-15

**Authors:** Kexin Feng, Shuangtao Zhao, Qingyao Shang, Guangdong Qiao, Jiaxiang Liu, Chenxuan Yang, Ya Wei, Yalun Li, Fei Ren, Lixue Xuan, Xiang Wang, Xin Wang

**Affiliations:** 1 Department of Breast Surgical Oncology, National Cancer Center/National Clinical Research Center for Cancer/Cancer Hospital, Chinese Academy of Medical Sciences and Peking Union Medical College, Beijing, 100021 People’s Republic of China; 2 Department of Thoracic Surgery, Beijing Tuberculosis and Thoracic Tumor Research Institute/Beijing Chest Hospital, Capital Medical University, Beijing, 101149 China; 3 Department of Breast Surgery, Yantai Yuhuangding Hospital, Yantai, 264000 China; 4 Department of Breast Surgery, An Yang Tumor Hospital, An Yang, 455001 Henan China

**Keywords:** Primary breast lymphoma (PBL), IPI, R-IPI, NCCN-IPI, Rituximab, Breast cancer

## Abstract

**Background:**

Due to the rarity of PBL and the lack of large-scale studies, the prognostic value of IPI in PBL was controversial. Especially in the rituximab era, the ability of IPI to stratify prognosis in patients receiving immunochemotherapy was severely reduced. Then revised IPI (R-IPI) and National Comprehensive Cancer Network IPI (NCCN-IPI) were introduced. The present study aimed to evaluate the prognostic value of IPI and the other IPIs in patients with PBL in a Chinese population.

**Methods:**

We performed a multicenter retrospective study of 71 patients with PBL from 3 institutions in China. The Kaplan–Meier method and log-rank tests were used for the survival analysis. Cox regression analysis was performed to evaluate the prognostic factors. Subgroup analysis was performed to assess the prognostic significance of IPI scores, R-IPI scores, and NCCN-IPI scores.

**Results:**

The median follow-up was 4.7 years (0.7–21.8 years). The 5-year progression-free survival (PFS) and overall survival (OS) rates were 90.2% and 96.3%. In the multivariate analysis, only IPI scores and radiotherapy were significantly associated with OS and PFS (*P* < 0.05). Applying the R-IPI in our patient cohort indicates a significant difference in PFS between the two groups of R-IPI (*P* = 0.034) but not for OS (*P* = 0.072). And the NCCN-IPI was prognostic for OS (*P* = 0.025) but not for PFS (*P* = 0.066). Subgroup analyses of IPI showed that survival analysis of IPI scores for the PFS and OS of patients using rituximab were not significantly different (*P* > 0.05).

**Conclusions:**

Our study confirms the prognostic value of IPI in patients with PBL, but the predictive value of IPI proved to be relatively low with the addition of the rituximab. The R-IPI and NCCN-IPI can accurately assess the high and low-risk groups of PBL patients but were insufficient to evaluate the intermediate risk group.

**Supplementary Information:**

The online version contains supplementary material available at 10.1186/s12935-022-02772-y.

## Background

Primary breast lymphoma (PBL) is a relatively rare extranodal lymphoma due to the breast's negligible amount of lymphoid tissue. The definition was malignant lymphoma limited to the breast or the breast and ipsilateral axillary lymph nodes but without the concurrent disseminated disease [1]. A strong case can also be made to include patients with the involvement of regional (supraclavicular and internal mammary) nodes [1]. It represents 0.5% of all primary malignant neoplasms of the breast and less than 3% of extranodal lymphomas [2, 3]. Primary breast diffuse large B-cell lymphoma (PB-DLBCL) was the most common histologic subtype, accounting for 50–70% of all subtypes of PBL [4, 5]. PBL was associated with an inferior prognosis, with a 5-year survival rate of 46–61% and a high recurrence rate locally in the breast and the central nervous system (CNS) [6–9]. By Han’s classification [10], DLBCL can be clinically divided into germinal center B-cell-like lymphoma (GCB) and non-GBC types. The non-GCB subtype was identified prospectively as more aggressive and usually associated with poor prognosis in nodal DLBCL. In contrast to the general data on nodal DLBCL, there is a striking predominance of the non-GCB type in PB-DLBCL, and the predictive value of GCB classification is controversial. Currently, the GCB classification and the IPI score are the two most commonly used prognostic factors for DLBCL patients. The International Prognostic Index (IPI) score was initially designed for risk stratification in aggressive lymphomas in the pre-rituximab era. Still, its prognostic value is challenged in the rituximab treatment era. The IPI score includes five clinical parameters (age, ECOG score, clinical staging, LDH levels, and the number of sites of extranodal invasion). Then a revised IPI (R-IPI) [11] has been introduced, which is a redistribution of the original IPI factors, and may serve as a simplified and more accurate predictor of the outcome of aggressive lymphoma patients treated with standard immunochemotherapy. An advanced IPI (National Comprehensive Cancer Network NCCN-IPI) was recently published by Zhou et al. [12]. By readjusting the age, LDH ratio, and extranodal disease, the NCCN‐IPI showed better discrimination of patient outcomes (both overall survival and PFS) than the original IPI.

Due to the clinical and biological heterogeneity of PBL and a small number of reported cases, the predictive value of IPI and other updated versions in PBL were controversial, and no cohort with a large size of patients to explore it [13–16]. It is crucial to identify the patients at high risk of relapse and select proper treatment strategies for them. Since the prognostic value of IPI is limited, more comprehensive prognostic indicators were constructed [17–21]. K T Troppan et al. propose modified IPI versions for more accurate prognostication and anemia [20, 21] and high C-reactive protein [17, 21], elevated uric acid levels [18], albumin and β2-microglobulin levels [19] can be used as indicators of poor prognosis of DLBCL. However, these prognostic indicators have not been recognized in the PBL.

Currently, most reports of PBL are from Western countries or regions, with a scarcity of data reported for Chinese patients. In this study, we screened out the predictive value of IPI is currently controversial.

Through a thorough literature review, and the statistical analyses used meta-analysis. To explore the predictive value of IPI and other updated versions in PBL in the Chinese cohort, a multicenter real-world retrospective study of 71 patients with PBL from three Chinese medical centers was performed. We retrospectively evaluated the clinical characteristics, immunophenotypes, treatment strategies, and prognostic factors of these 71 patients.

## Methods

### Patients

The study cohort consisted of 71 PBL patients treated at three medical centers in China, including the Cancer Hospital Chinese Academy of Medical Sciences, Yantai Yuhuangding Hospital, and Anyang Tumor Hospital between 2000 and 2020. Patients were excluded if they (1) had a primary site that was difficult to determine; (2) had breast involvement secondary to systemic disease; (3) had a disease that had histologically transformed from low-grade lymphoma. None of these patients underwent preoperative chemotherapy or radiation therapy. An initial evaluation of all patients included a history and physical examination, complete blood counts, and chemistries. Computed tomography (CT) of the chest, abdomen, and pelvis was done. Due to the study time duration, other imaging studies, such as positron emission tomography (PET) scans, were variable. Patients were diagnosed and treated at the Department of Breast Surgery or the respective institutions in China. All clinicopathological data were retrieved from medical records and pathology reports of the Institute of Pathology from the individual institutions. Diagnostic tissue biopsies were obtained from the breast mass by a core needle biopsy, excisional biopsy, and mastectomy. Pathological diagnoses were based on hematoxylin and eosin (HE) stained slides, and the classification was based on the WHO classification [22].

Patients were staged according to the Ann Arbor system. The stages IE were restricted to the breast, and stages IIE involved regional axillary lymph nodes. Patients with bilateral breast involvement were classified as stage IV in this study. The IPI was determined using all the available information.

Age, sex, treatment modality, stage, tumor size, Hans classification, IPI, Ki-67, B-cell lymphoma (Bcl)2, BCL6, c-Myelocytomatosis oncogene (c-Myc) protein expression, B symptom, overall survival status, and specific routine blood tests, and biochemical examination findings were evaluated. The leukocyte count (WBC), neutrophil (Neut) counts, and lymphocyte (Lmp) measures in the pretherapeutic blood routine of these patients were collected. Biochemical markers studied were β2-microglobulin (β2-MG) and serum lactate dehydrogenase (LDH), all obtained by pre-diagnosis exploration 1–7 days before histological diagnosis was obtained.

The expression of cluster of differentiation (CD)5, CD30, c-Myc in tumor samples from 71 patients using immunohistochemistry assay (IHC). IHC for CD5 was judged as positive if  > 30% of tumor cells were stained and 20% for CD30. P53 positive staining was defined as positive nuclear staining. For c-Myc, staining of the nucleus in more than 40% of the positive tumor cells was regarded as positive. The expression of the EBV encoded RNA (EBER) encoded by the Epstein-Barr virus (EBV) was detected using fluorescence in situ hybridization (FISH). Diffuse staining greater than 70% of cells was considered positive for BCL-6 and BCL-2. Double-hit lymphoma is defined as high-grade B-cell lymphoma with c-Myc and BCL-2 or BCL6 rearrangements, and triple‐hit lymphoma is composed of B‐cell lymphoma with translocation of c-Myc, BCL-2, and BCL6.

Follow-up started at the time of surgery. The primary clinical information is listed in Table 1. As 45 patients (63.4%) were low-risk group (IPI scores 0–1) IPI scores, and the other 36.6% patients were low-mediate-risk to mediate-high- to high-risk groups (IPI scores 2–5), we stratified patients with PBL into IPI scores 0–1 group and IPI scores 2–5 group.

**Table 1:** Clinical characteristics of the 71 patients evaluated, categorized by IPI scores

	Total (n = 71)	IPI 0–1 (n = 45)	IPI 2–5 (n = 26)	P**
Age, years				0.592^†^
< 60	40	34	6	
≥ 60	31	11	20	
Follow-up duration				
Median years (range)	4.7 (0.7–21.8)	4.1 (1.0–21.8)	4.8 (0.7–14.6)	0.025^*^
Laterality				0.614^†^
Right	42	29	13	
Left	23	12	11	
Bilateral	6	4	2	
Nodal site involvement at diagnosis				0.348^†^
None	45	31	14	
Axillary ± supraclavicular	26	14	12	
Presence of B-symptoms				NA
Absent	71	45	26	
Present	0	0	0	
Pathological classification				0.513^†^
DLBCL	57	35	22	
Others	14	10	4	
Pathology				0.315^†^
Non-GCB	29	21	8	
GCB	23	14	9	
Unknown	5			
Ann Arbor stage				0.342^†^
IE	39	27	12	
IIE	26	14	12	
IVE	6	4	2	
Tumor Size in BUS1				0.047^†^
≥ 5 cm	7	2	5	
< 5 cm	64	43	21	
LDH				0.009^†^
Normal	34	29	5 11	
Elevated	26	12	14	
Unknown	11	4	7	
β2-MG				0.541^†^
Normal	37	25	12	
Elevated	17	10	7	
Unknown	17	10	7	
WBC				0.855^†^
Normal	61	37	24	
Elevated	3	2	1	
Unknown	7	6	1	
Neut				0.622^†^
Normal	38	24	14	
Elevated	25	17	8	
Unknown	8	4	4	
Lymph				0.894^†^
Normal	37	22	15	
Elevated	16	10	6	
Unknown	18	13	5	
Ki-67 (%)				0.205^†^
< 50%	11	5	6	
≥ 50%	57	37	20	
Unknown	3	3	0	
BCL-2				0.333^†^
Positive	40	24	16	
Negative	25	17	8	
Unknown	6	4	2	
BCL-6				0.535^†^
Positive	34	20	14	
Negative	10	7	3	
Unknown	27	18	9	
c-Myc				0.990^†^
Positive	23	17	6	
Negative	26	15	11	
Unknown	22	13	9	
Double-hit-lymphomas				0.333^†^
Yes	23	15	8	
No	26	17	9	
Unknown	22	13	9	
Triple-hit-lymphomas				
Yes	16	8	8	0.217^†^
No	16	11	5	
Unknown	39	26	13	
P53				0.556^†^
Positive	23	15	8	
Negative	23	12	11	
Unknown	25	18	7	
CD30				0.936^†^
Positive	14	9	5	
Negative	27	17	10	
Unknown	30	19	11	
CD5				0.910^†^
Positive	19	11	8	
Negative	32	19	13	
Unknown	20	15	5	
EBER				0.094^†^
Positive	7	6	1	
Negative	51	33	18	
Unknown	13	6	7	
Surgery				0.117^†^
Yes	44	30	14	
No	27	15	12	
Circles of Chemotherapy				0.947^†^
< 4	2	1	1	
≥ 4	51	32	19	
Unknown	18	12	6	
Rituximab Given				0.033^†^
Yes	42	26	16	
No	13	4	9	
Unknown	16	15	1	
RT				0.618^†^
Yes	9	7	2	
No	17	11	6	
Unknown	45	27	18	
CNS prophylaxis administered				0.005^†^
Yes	6	3	3	
No or unknown	65	42	23	

For bilateral cases, this is the larger value of the left and right breast diameters

*BUS* breast ultrasound, *IPI* International Prognostic Index, *DLBCL* diffuse large B-cell lymphoma, *GCB* germinal center B-cell like, *LDH* lactate dehydrogenase, *β2-MG* β2-microglobulin, *WBC* white blood cell, *Neut* neutrophils, *Lymph* lymphocytes, *BCL* B-cell lymphoma, *CD* cluster of differentiation, *RT* radiotherapy, *CNS* central nervous system, *NA* not available

^**^p indicates a *p*-value between two groups, “IPI 0–1” and “IPI 2–5”

^*^Mann–Whitney-U test

^†^Pearson's Chi-square test

### Statistical analysis

The final follow-up was done on December 1, 2021. Overall survival (OS) was calculated from the time of surgical intervention to the date of last follow-up or death from any cause. In contrast, PFS was calculated from the date of surgical intervention to the date of first recurrence and progression or death for any reason. The recurrence was defined as the pathologically confirmed recurrence of recurrent disease in the same breast or regional lymph nodes following the completion of definitive therapy.

The survival analysis of OS and PFS were analyzed using the Kaplan–Meier method. The univariate analysis was performed to evaluate the prognostic factors of patients with PBL in our cohorts. Prognostic factors (*P* < 0.1) in univariate analysis were subjected to the Cox proportional-hazards model for multivariate survival analysis, and the hazard ratios (HRs) and 95% confidence interval (CI) were obtained by the Cox proportional hazards regression model to determine the simultaneous impact of prognostic factors on survival. The chi-squared (χ2) test was performed to compare clinical characteristics, and differences were tested using the two-tailed test. *P* < 0.05 was considered statistically significant. In this study, all visualizations were generated by the R language (version 4.1.2).

## Results

**Fig. 1:**
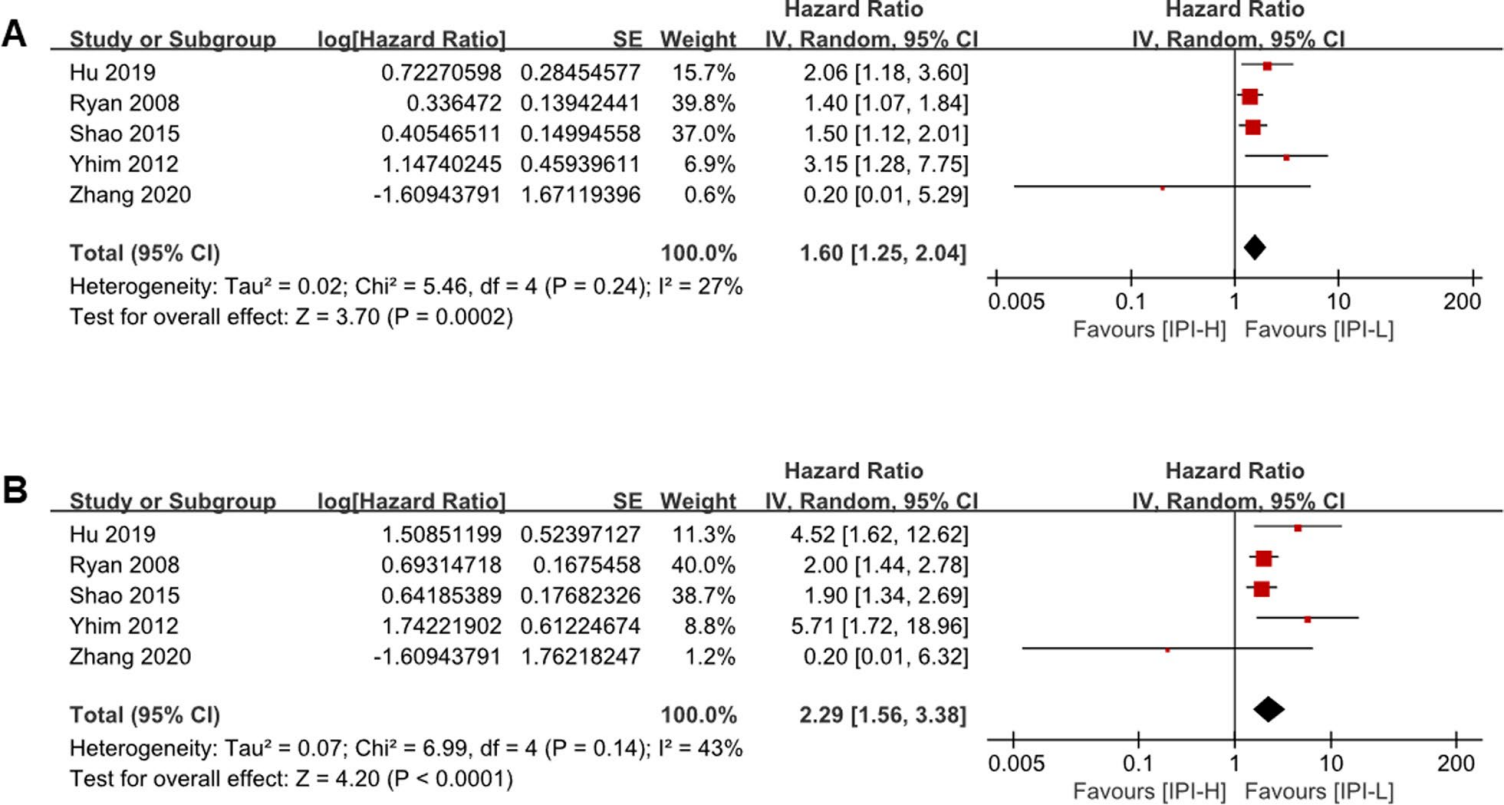
Forest plot of hazard ratio (HR) of IPI scores for progression-free survival (PFS) and overall survival (OS). **A**, Forest plot of HR of IPI scores for PFS; **B**, Forest plot of HR of IPI scores for OS

We reviewed current literature between its' first report of PBL in January 1975, and June 2020 performed using PubMed and Web of Science databases. The terms “primary breast lymphoma [Title]) OR (primary lymphoma of breast [Title]” were used as keywords for database searches. Non-related studies and studies not published in English were excluded from this analysis. Studies with fewer than 20 cases were excluded from the analysis. The results regarding IPI scores were presented in the forest graph format (Fig. 1). The forest plot showed that IPI 2–5 was significantly associated with worse OS and PFS (pooled HR 1.60, 95% CI 1.25–2.04; pooled HR 2.29, 95% CI 1.56–3.38; respectively, Fig. 1).

### Baseline characteristics

Totally 71 patients were enrolled in this study. The baseline characteristics of the 71 enrolled patients are presented in Table 1. The median age was 57 years (23–86 years). Fifty-one patients (71.8%) presented with painless palpable breast masses. The right breast was more likely to be involved than the left breast (59.1% vs. 32.4%). Six patients (8.5%) presented with bilateral breast involvement. No patients had any clinical B symptoms (fever, weight loss, and night sweats). The median tumor size by BUS was 2.8 cm (range, 0.4–5.3 cm), with seven (9.9%) patients having a tumor of more than 5 cm. 18F-FDG PET/CT is widely used in DLBCL, mainly including staging, aggressiveness, prognosis, and efficacy monitoring. The median maximum standardized uptake value (SUVmax) of PBL patients was 9.2, and the range of values is extensive (1.4–26.6). Twenty-three patients (32.4%) had axillary lymph node involvement at the initial diagnosis. Thirty-four patients (47.9%) had elevated LDH; 17 patients (23.9%) had elevated β2-MG; three patients (4.2%), 25 patients (35.2%) and 16 patients (22.5%) had elevated number of WBC, neut and lymph, respectively.

### Pathological characteristics

Among 71 patients, 57 patients (80.3%) were DLBCL subtype, four patients were marginal zone lymphomas/ mucosa-associated lymphoid tissue (MZL/MALT), and three patients were follicular lymphoma (FL), two patients were T cell lymphoma. Only one patient was mantle cell lymphoma (MCL). The pathologic subtypes did not have significant differences in the two groups (*P* = 0.513, Table 1). Moreover, by univariate and multivariate analysis in patients with PBL, the pathologic subtypes were not an independent prognostic factor affecting survival (*P* = 0.671 for OS, *P* = 0.781 for PFS; Table 2); we did not consider the effect of these non-DLBCL pathologic subtypes.

**Table 2:** Univariate and multivariate analysis of the survival of patients with PBL

Variables	Cases, n	Overall survival				Progression-free survival			
		Univariate analysis		Multivariate analysis		Univariate analysis		Multivariate analysis	
		*P*-value	HR (95% CI)	*P*-value	HR (95% CI)	*P*-value	HR (95% CI)	*P*-value	HR (95% CI)
Age, years		**0.052**	5.243 (0.985–27.899)	0.061	4.621 (1.032–41.781)	**0.049**	3.169 (1.008–9.967)	0.882	1.150 (0.182–7.264)
< 60	40								
≥ 60	31								
Ann arbor stage		0.402	0.708 (0.218–2.302)			0.844	0.937 (0.488–1.797)		
IE	39								
IIE	26								
IVE	6								
IPI		**0.029**	11.945 (1.281–111.394)	0.046	6.474 (1.021–41.145)	**0.003**	7.918 (1.993–31.454)	0.045	6.386 (1.043–39.090)
0–1	45								
2–5	26								
Pathological classification		0.671	0.637 (0.045–6.224)			0.781	0.653 (0.256–3.790)		
DLBCL	57								
Others	14								
Pathology		0.796	0.739 (0.075–7.269)			0.800	0.808 (0.155–4.215)		
Non-GCB	29								
GCB	23								
LDH		**0.056**	5.109 (0.958–27.259)	0.853	1.305 (0.078–21.773)	**0.031**	3.675 (1.075–12.566)	0.213	2.833 (0.549–14.616)
Normal	34								
Elevated	26								
β2-MG		0.384	2.413 (0.333–17.505)			0.758	1.245 (0.309–5.012)		
Normal	23								
Elevated	29								
WBC		0.515	2.044 (0.237–17.632)			0.999	1.001 (0.128–7.841)		
Normal	61								
Elevated	3								
Neut		0.993	0.993 (0.211–4.676)			0.532	0.699 (0.227–2.151)		
Normal									
Elevated									
Lymph		0.684	0.632 (0.069–5.773)			0.752	1.252 (0.310–5.060)		
Normal	34								
Elevated	26								
Ki-67 (%)		0.647	1.651 (0.192–14.172)			0.573	1.555 (0.335–7.220)		
< 50%	11								
≥ 50%	57								
c-Myc		0.942	1.032 (0.444–2.401)			0.22	1.1429 (0.807–2.529)		
Positive	23								
Negative	26								
BCL-2		**0.063**	6.237 (0.905–42.995)	0.091	6.980 (0.639–45.946)	**0.022**	4.636 (1.246–17.255)	0.709	1.521 (0.168–13.729)
Positive	40								
Negative	25								
BCL-6		**0.003**	0.082 (0.015–0.431)	0.118	0.237 (0.039–1.439)	**0.006**	0.242 (0.088–0.670)	0.856	0.860 (0.169–4.379)
Positive	34								
Negative	10								
Double -hit-lymphomas		0.654	0.801 (0.213–2.008)			0.844	0.876 (0.479–1.135)		
Yes	23								
No	26								
Unknown	22								
Triple-hit- lymphomas		0.821	0.791 (0.158–2.548)			0.701	0.860 (0.178–2.901)		
Yes	16								
No	16								
Unknown	39								
P53		0.947	1.026 (0.483–2.178)			0.821	0.938 (0.538–1.635)		
Positive	23								
Negative	23								
CD30		0.858	0.909 (0.321–2.579)			0.236	0.670 (0.345–1.300)		
Positive	14								
Negative	27								
CD5		**0.088**	0.254 (0.053–1.226)	0.326	0.403 (0.066–2.470)	**0.017**	0.361 (0.156–0.834)	0.365	0.511 (0.119–2.187)
Positive	19								
Negative	32								
EBER		0.639	0.410 (0.080–5.735)			0.972	0.963 (0.120–7.725)		
Positive	7								
Negative	51								
Surgery		0.933	1.102 (0.115–10.540)			0.643	0.721 (0.180–2.878)		
Yes	44								
No	27								
Rituximab given		**0.092**	2.632 (0.158–43.713)	0.549	2.907 (0.089–95.347)	**0.091**	2.602 (0.183–9.914)	0.702	0.850 (0.345–2.094)
Yes	42								
No	13								
RT		**0.087**	0.708 (0.267–1.877)	0.047	1.385 (0.201–9.550)	**0.061**	0.516 (0.258–1.031)	0.042	1.409 (0.024–8.148)
Yes	9								
No	17								

Boldface values in the table indicate* P* < 0.1 in univariate analysis and* P* < 0.05 in multivariate analysis

*HR* Hazard ratio, *CI* Confidence interval, *IPI* International Prognostic Index, *GCB* germinal center B-cell like, *LDH* lactate dehydrogenase, *β2-MG* β2-microglobulin, *WBC* white blood cell, *Neut* neutrophils, *Lymph* lymphocytes, *BCL* B-cell lymphoma, *CD* cluster of differentiation, *RT* radiotherapy

**Fig. 2:**
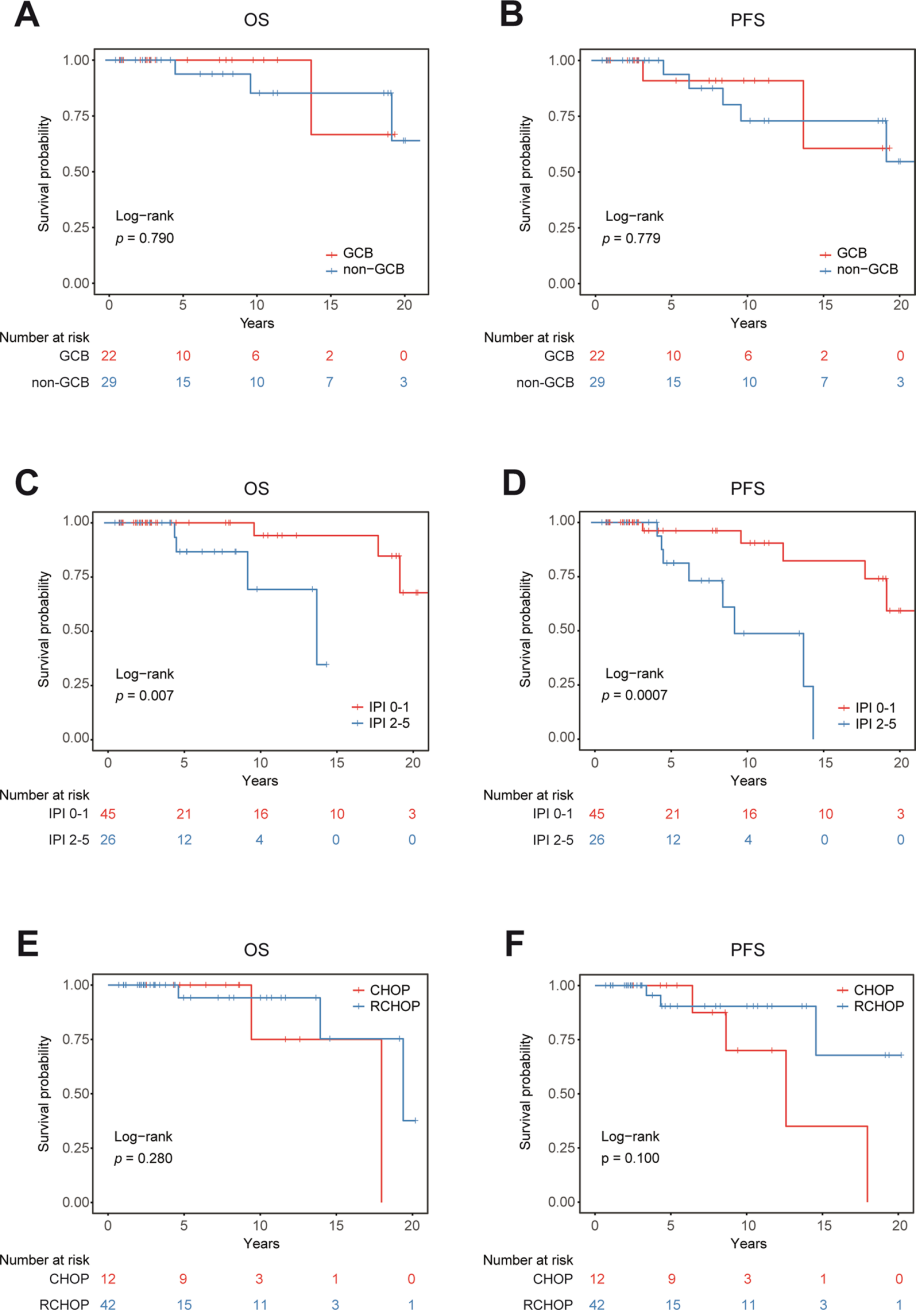
Kaplan–Meier survival curves of patients with PBL. **A**, Kaplan–Meier curve of OS for patients of GCB and non-GCB; **B**, Kaplan–Meier curve of PFS for patients of GCB and non-GCB; **C**, Kaplan–Meier survival curves for OS of patients with IPI of 0–1 and 2–5; **D**, Kaplan–Meier survival curves for PFS of patients with IPI of 0–1 and 2–5; **E**, Kaplan–Meier survival curves for OS of patients treated with CHOP and RCHOP; **F**, Kaplan–Meier survival curves for PFS of patients treated with CHOP and RCHOP

Among the patients with PB-DLBCL, 23 patients (32.4%) were classified as GCB phenotype and 29 patients (40.8%) as non-GCB type according to the algorithms described by Hans et al.[10]. No statistically significant difference in PFS and OS was found between patients with GCB phenotype and patients with the non-GCB type (Fig. 2A, B). The BCL-2 expression was positive in 40 (56.3%) patients, BCL-6 expression in 34 (47.9%) patients, c-Myc expression in 23 (32.4%) patients, c-Myc/Bcl-2 protein co-expression in 23 (32.4%) patients, CD30 expression in 14 (19.7%) patients, CD5 expression in 19 (26.7%), and P53 expression in 23 (32.4%) patients. The EBER expression in seven (9.9%) patients. Among 68 patients with a record of Ki-67, a proliferation index of Ki-67 < 50% was found in 11 patients, and Ki-67 > 70% in 57 patients.

### Staging and risk stratification

Based on the Ann Arbor clinical staging criteria, of the 71 patients, 39 patients (54.9%) were stage IE, 26 patients (36.6%) were stage IIE, and 6 patients with bilateral breast masses were stage IV. Risk stratification was investigated based on IPI scores; 45 patients (63.4%) had 0–1 IPI score, 16 patients (22.5%) had IPI scores of 2; eight patients (11.3%) were 3, and two patients (2.8%) 4–5. As we stratified patients with PBL into IPI scores 0–1 group and IPI scores 2–5 group, there was a statistically significant difference in PFS (*P* = 0.007, HR 0.184, 95%CI 0.029–1.185) and OS (*P* = 0.0007, HR 0.199, 95%CI 0.056–0.709) between these two groups (Fig. 2C, D). The median time to OS endpoint in the IPI 0–1 group and IPI 2–5 groups were not reached, and the median time to PFS in these two groups was 13.9 years. The present study demonstrated that IPI applies to PBL patients as a prognostic factor for predicting both OS and PFS.

### Treatment

Overall, 43 (60.6%) patients were diagnosed by surgical biopsy, and 28 (39.4%) underwent core needle biopsy. A total of 44 (62.0%) patients received breast surgery (40 lumpectomies, a straightforward mastectomy, and three modified radical mastectomies). The surgery rates in these three hospitals were 64.1%, 40.0%, and 100.0%, respectively. Eighteen patients have not recorded chemotherapy cycles, regimens, and doses and are not addressed here. Two patients refused chemotherapy, and a total of 54 patients received chemotherapy. Regarding chemotherapy regimens, 54 patients received cyclophosphamide, doxorubicin, vincristine, and prednisone (CHOP), whereas 42 received rituximab (R) plus CHOP (RCHOP). The fifty-one (71.8%) patients completed four cycles of chemotherapy, and the median number of chemotherapy cycles completed was six. No statistically significant difference in PFS and OS was found with the addition of rituximab in chemotherapy of PBL (Fig. 2E, F).

Except for 45 (63.4%) patients who were not recorded, nine received chemotherapy combined with radiotherapy (RT), and 17 did not receive RT. The area of elective-field radiotherapy included the uninvolved breast and supra-/infraclavicular lymphatics, as well as the axillary lymphatics. The radiotherapy dose ranged from 30 to 40 Gmy (Gy) with a median value of 32 Gy. The CNS prophylaxis was administered in six (8.4%) patients.

### Prognostic impact of the R-IPI and NCCN-IPI

**Fig. 3:**
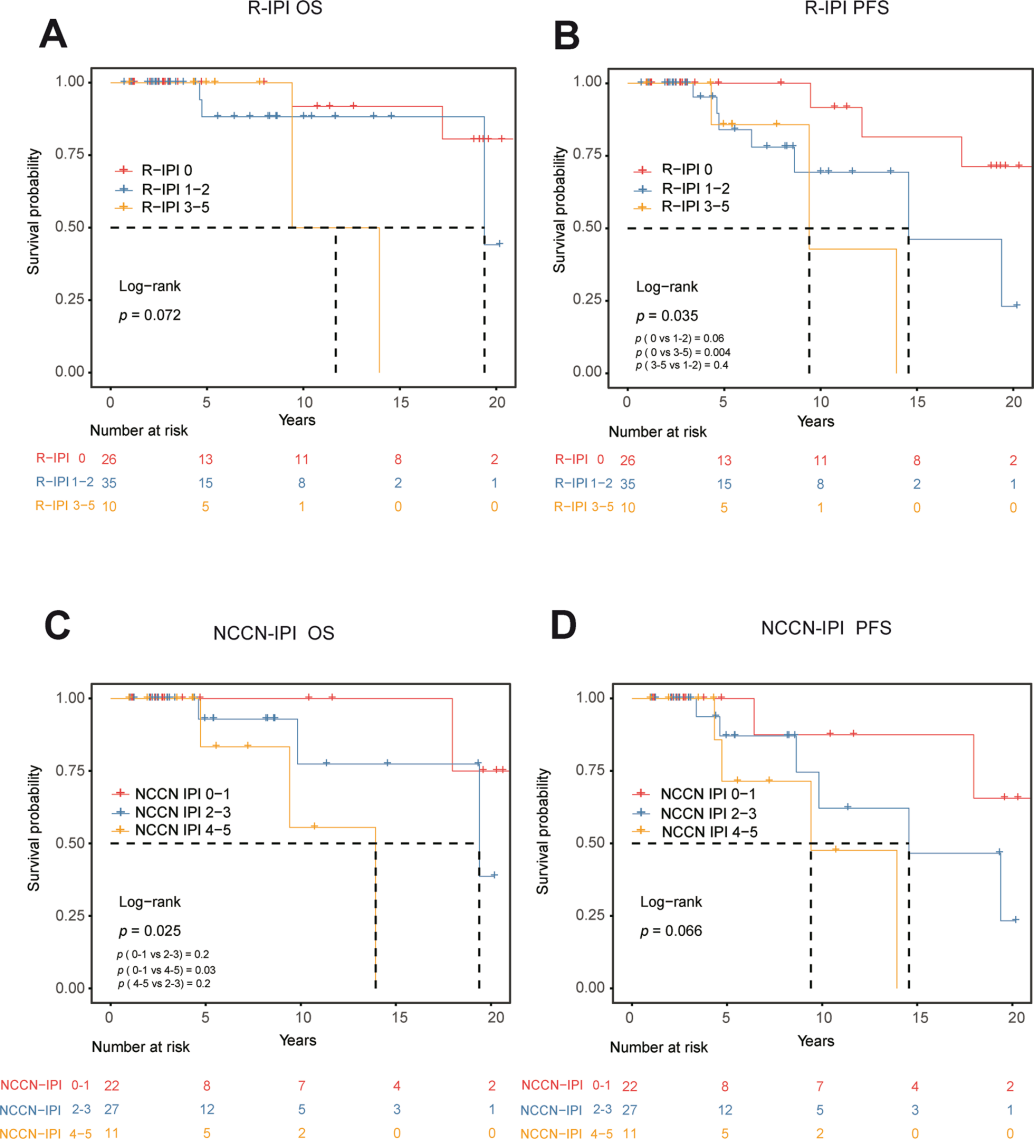
Kaplan–Meier survival curves of patients with PBL categorized by R-IPI scores and NCCN-IPI scores. **A**, Kaplan–Meier survival curves for OS of patients with R-IPI scores; **B**, Kaplan–Meier survival curves for PFS of patients with R-IPI scores; **C**, Kaplan–Meier survival curves for OS of patients with NCCN-IPI scores; **D**, Kaplan–Meier survival curves for PFS of patients with NCCN-IPI scores

Applying the R-IPI in our patient cohort resulted in 26 (36.6%) patients classified as very good risk (R-IPI 0), 35 (49.3%) patients as good risk (R-IPI 1–2), and ten (14.1%) patients as a poor risk. The difference in the OS rates was insignificant among these three groups (*P* = 0.072). For PFS outcomes, the R-IPI identified three prognostic groups of patients with highly different (5-year PFS: 100.0%, 87.5%, 84.0%, respectively; *P* = 0.034, Fig. 3A). However, it is noteworthy that only the PFS outcome between R-IPI 0 and R-IPI 3–5 was significant (*P* = 0.004, Fig. 3B). The curves of R-IPI 0 versus R-IPI 1–2 and R-IPI 1–2 versus R-IPI 3–5 were not significant (*P* = 0.06 and *P* = 0.4, respectively; Fig. 3B). This result indicates that the R-IPI can accurately assess the high and low-risk groups but was insufficient to assess the intermediate-risk group.

Then we also applied the NCCN-IPI to our patient cohort. We removed 11 patients with incomplete clinical data and classified them into three groups. Twenty-two patients were low-risk group (NCCN-IPI 0–1), 27 patients were intermediate-low risk group (NCCN-IPI 2–3), and 11 patients were medium–high risk group (NCCN-IPI 4–5). No patients were in the high-risk group (NCCN 6–8). The difference in the OS rates was significant among these three groups (*P* = 0.025; Fig. 3C). Same as the R-IPI, the NCCN-IPI only has advantages in assessing the high and low-risk groups; only the curve of NCCN-IPI 0–1 versus NCCN-IPI 4–5 was significant (*P* = 0.03; Fig. 3C). The difference in the PFS rates was insignificant among these three groups (*P* = 0.066; Fig. 3D). This result suggested that the NCCN-IPI was also insufficient to assess the intermediate risk group.

### Follow-up and prognosis

Until the end of follow-up, all patients in the active follow-up phase had at least 6 months of follow-up. The median follow-up was 4.7 years (0.7–21.8 years). For the 54 patients received CHOP, 18 (34.0%) patients got complete regression (CR) after first treatment, ten (18.9%) patients got partial response (PR), 14 (26.4%) patients presented as stable disease (SD), while the other 11 (20.8%) patients developed progression (PD) following first-line therapy. Of the 18 patients who got CR after first treatment, seven (38.9%) patients developed PD following first-line therapy, with four patients having more than two sites of involvement at first progression. The breast was a site of first progression in three patients, regional nodes in two, CNS in three, and lumbar in one. A total of five patients died. The 5-year PFS and OS rates were 90.2% and 96.3%, while the 10-year PFS and OS rates were 75.3% and 89.9%, respectively. The rate of CNS relapse was 4.2%. The median and average time to relapse were 13.0 and 27.8 months, respectively, and five (71.4%) patients relapse within 2 years. Among the seven patients with recurrence, two were MZL/MALT subtypes, and the others were all DLBCL subtypes.

### Correlation analysis of clinical characteristics and treatment methods with PFS and OS rates

**Fig. 4:**
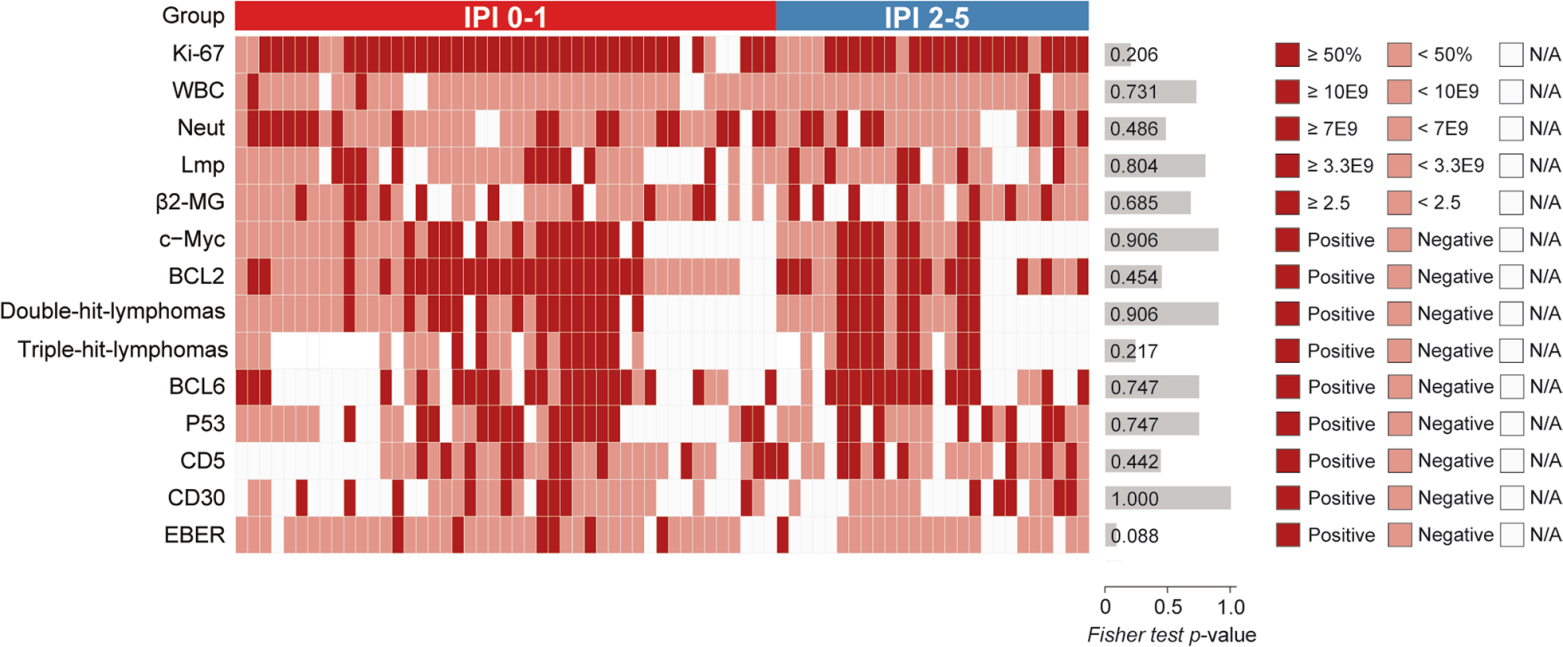
Heatmap of the pathologic and immunohistochemical characteristics between PBL patients in IPI 0–1 and IPI 2–5 groups. *WBC* white blood cell, *Neut* neutrophils, *Lymph* lymphocytes, *β2-MG* β2-microglobulin, *BCL* B-cell lymphoma, *CD* cluster of differentiation, *EBER* Epstein-Barr virus encoded RNA

The pathologic and immunohistochemical characteristics of PBL patients in IPI 0–1 and IPI 2–5 groups were not significantly different between the two groups (*P* > 0.05, Fig. 4), which indicates that the pathologic and immunohistochemical characteristics were not associated with survival outcomes.

Univariate analysis was performed for patients' age, Ann Arbor stage, IPI score, Hans classification, serum LDH level, serum β2-MG level, serum WBC level, serum neutrophils, and lymph lymphocytes level; Ki-67; BCL-2 protein, BCL6 protein, P53, CD30, CD5 and c-Myc protein expression level, surgery, administration of rituximab and radiotherapy, as listed in Table 2. The results showed that patients’ age, IPI score, serum LDH level, BCL-2 protein expression level, administration of rituximab, and radiotherapy were significantly associated with OS (*P* < 0.1), while in the multivariate analysis, only IPI scores and radiotherapy were significantly associated with OS (*P* < 0.05). As for PFS, in the univariate analysis, the patients' age, IPI score, serum LDH level, BCL-2 protein, BCL-6 protein and CD5 protein expression level, administration of rituximab, and radiotherapy were risk factors (*P* < 0.1). In the multivariate analysis, the IPI scores and RT were risk factors for PFS (*P* < 0.05). Though double-hit-lymphomas and triple-hit-lymphomas are clinically aggressive neoplasms with poor prognosis, no significant clinical influence of the immunohistochemical assessment of c-Myc and BCL-2/BCL-6 was found in multivariate analyses.

Moreover, we made ROC curves to assess the sensitivity and specificity for 5-year survival of IPI and other predictive factors. Compared with other predictive factors, which were significant in univariate analysis, the IPI had the largest AUC (0.8333 ROC curves of OS; 0.8088 ROC curves of PFS, Additional file 3: Figure S1). As a clinical predictive factor, compared with other clinical characteristics, IPI is more accurate.

The clinical data of each patient were in Additional file 1: Table S1.

### Subgroup analysis of IPI in patients treated with rituximab

To further explore the prognostic significance of the IPI scores, we divided 71 patients into two groups, and a subgroup analysis was conducted. In the two groups of patients with different IPI scores, the administration of rituximab is significantly different, as indicated (*P* = 0.033). Survival analysis of IPI scores was performed in patients grouped as RCHOP and CHOP. Consistent with the results of ungrouped data, the Kaplan–Meier survival curves of the IPI scores for the PFS and OS of patients in the RCHOP group were not significantly different (*P* > 0.05). (Additional file 4: Figure S2A, B). No significant differences were observed between survival curves for OS in the CHOP group (*P* = 0.083; Additional file 4: Figure S2C). However, in the CHOP group, significant differences were found in the Kaplan–Meier survival curves for the PFS (*P* = 0.034; Additional file 4: Figure S2D).

### Basic information of studies included in the literature review

The flowchart of the literature search is shown in Additional file 5: Figure S3. The basic information of 25 studies was listed in the Additional file 2: Table S2. The number of participants ranged from 11 to 465 in every cohort study and the follow-up period of the cohorts ranged from 2.7 to 8.0 years. The ages were mainly younger than 60 years old, with the median ages ranging from 43 to 67 years old. Among these 25 articles, only 3 were on non-DLBCL subtypes of PBL. Apart from 8 articles without a record on the onset site, 13 articles recorded the right breast as the leading onset site, and 12 articles had a history of bilateral PBL. The 5-year OS rates ranged from 36.2 to 93.5%, and the 5-year PFS rates ranged from 29.1 to 95.4%. Twelve articles reported CNS relapse, ranging from 5 to 28.3%.

## Discussion

The median age at diagnosis in Western countries is 60–64 years [4, 23]; in East Asian countries, the median age is lower (40–60 years) [5]. Our study's median age was 57 years (range 23–86 years), consistent with other reports.

This study investigated the association between pathological features and prognosis in patients with PBL. PB-DLBCL is the most common histologic subtype of PBL, comprising 40–80% [4, 16, 24, 25]. MZL (9–28%) and MALT lymphoma (12.2%) are the next most frequent [1, 4, 26, 27]. FL (10–19%)[1, 26, 28] and Burkitt lymphoma (1–5%) [29, 30] are also identified in the breast but have a lower prevalence [4]. In this study, PB-DLBCL accounts for a major portion (80.3%), followed by MZL/MALT (5.6%) and FL (4.2%). Among the seven patients with recurrence, two were MZL/MALT subtypes, and the others were all DLBCL subtypes.

By Han′s classification [10], DLBCL can be clinically divided into GCB and non-GBC types. Previous studies showed that most patients with PB-DLBCL are of the non-GCB, yet reported treatment outcomes were similar between the GCB and non­GCB patients. In line with the studies mentioned above, 63.8% of patients with PB-DLBCL were of the non-GCB type, accounting for most of these cases. Consistent with other studies, the 5-year OS and PFS in patients with GCB type were not significantly higher than those with the non-GCB type*.* Double- and triple-hit lymphomas are high-grade B-cell lymphomas and have been suggested to be prognostic factors in DLBCL but remain ambiguous in PBL. In our cohort, there was no apparent difference in outcomes for the few patients with double-hit lymphoma in this trial.

Though surgical intervention beyond excisional biopsy has been shown to offer no improvement in survival or recurrence risk [2, 8, 9, 31, 32], in the present study, 60.6% of all patients are diagnosed by surgery. Through the search of 27 studies, the ranges of rates of surgery were 4.4% [6] and -100.0% [33]. The surgery rates in these three hospitals were 64.1%, 40.0%, and 100.0%, respectively. One reason could be that most FNAC results only suggested but not definitively diagnosed PBL, and the second could be that some patients with palpable masses tend to undergo surgery.

By reviewing the relevant literature, we found the present clinical issue, that the prognostic value of IPI in PBL is controversial. Studies by Ryan et al. [6], Hu et al. [9], Zhang et al. [34], Shao et al. [15], Yhim et al. [35] demonstrated that the IPI scores were independent risk factors for PFS and OS, while results from Zhao et al. [36], Qu et al. [37] and Zhang et al. [38] confirmed that the IPI scores were not associated with PFS and OS in PBL. The result was presented in the forest graph format (Fig. 1). Then, a retrospective study of 71 patients were conducted. In this study, 45 (63.4%) patients had an IPI score of 0–1, and 26 (36.6%) patients had a high IPI score (of 2–5). The survival curves revealed low IPI scores (0–1) were significantly associated with PFS and OS. (Fig. 2C, D). The univariate and multivariate COX regression analysis illustrated that IPI scores were independent prognostic factors for PFS and OS (*P* < 0.05, Table 2). Moreover, the ROC curves assessing the sensitivity and specificity for 5-year survival of IPI and other predictive factors showed that the IPI had the largest AUC. The present study demonstrated that IPI applies to PBL patients.

Findings in our cohort by the subgroup analysis, in patients who did not use rituximab, IPI was proved to be a prognostic factor of the PFS (*P* = 0.034, Additional file 4: Figure S2D. The subgroup results suggested that in the patients without rituximab, IPI proved valuable in predicting the prognosis of PBL; however, IPI seems to have lost some of its predictive value with the addition of rituximab.

The R-IPI is a redistribution of the original IPI factors, which may serve as a more accurate predictor of outcome than the standard IPI. The NCCN-IPI risk score focuses on the involvement of defined high-risk extranodal sites, not on the number of involved extranodal sites, and includes the negative impact of very high LDH levels. Very few studies explored the prognostic value of R-IPI and NCCN-IPI in PBL. Applying the R-IPI and NCCN-IPI in our patient cohort indicates that the predictive values of R-IPI and NCCN-IPI are limited, especially suboptimal in high-risk patients.

In this study, only six patients received prophylactic intrathecal chemotherapy, which may be due to the controversial clinical evidence of its benefit. The results of large-scale retrospective studies have not found that prophylactic intrathecal chemotherapy can prevent the recurrence of CNS for PBL patients. Hosein et al. [8] summarized the data of 76 PBL patients from 8 centers in the United States with a follow-up of 10 years and found that the incidence of CNS recurrence in PBL patients was high. The incidence of CNS was not related to disease stage, LDH level, IPI score, and use of rituximab. Though none of these six patients had CNS infiltration, a correlation between prophylactic intrathecal chemotherapy and prognosis cannot be proven.

Several limitations need to be noted in this study. First, due to insufficient awareness in the early years, PET/CT was not widely used, especially in local hospitals. Not all patients are assessed for CNS involvement at diagnosis, which may lead to underestimation and inclusion of some patients with occult CNS disease. Due to the limited number of patients who received prophylactic intrathecal chemotherapy, we did not explore the preventive interventions for CNS. Further large-scale prospective clinical trials are needed to investigate how to prevent the CNS recurrence of PBL. Then, the low case number in the subgroups of patients with or without rituximab made our comparative result insufficient. However, as a real-world study, the development of subgroup analysis is still helpful in providing direction for future studies. Last, this study was retrospective, which is inevitably susceptible to selective, observational, and confounding bias. As the PBL has a low incidence, the epidemiology studies were difficult to conduct. We have added the geographic distribution of patients in Additional File 1: Table S1.

This study has several strengths, including the large sample size, long-term follow-up, and participating hospitals from different parts of the country. The other two hospitals are both regional medical centers with a large number of patients. Medical institutions are widely distributed in China, with a significant disparity in the medical levels of different regions, so it is necessary to consider differences in medical centers.

Due to the rarity of this disease, some multicenter and prospective trials are required to guide the clinical treatment of PBL in the future. Prognostic tools based on new clinical and molecular insights in the pathobiology of PBL are currently far from routine clinical application. Thus there is a relevant clinical need for more accurate prognostication. The next generation sequencing and cell experiments are required.

## Conclusions

In summary, this study comprehensively analyzes clinical features, patterns of failure, and the effect of various therapeutic strategies on PBL. Our study confirmed the excellent performance of the IPI in PBL and showed that it is prognostic, especially for patients without using rituximab. Additionally, the R-IPI and NCCN-IPI can be used to identify high-risk PBL patients for more intensive treatment.

## Supplementary Information

**Additional file 1: Table S1**.Clinical data of each patients.

**Additional file 2: Table S2.** Basic information of studies included in the literature review.

**Additional file 3: Figure S1. **ROC curves for 5-year survival of IPI and predictive factors significant in univariate analysis. A, ROC curves for 5-year OS of IPI and predictive factors significant in univariate analysis. B, ROC curves for 5-year PFS of IPI and predictive factors significant in univariate analysis.

**Additional file 4: Figure S2. **Kaplan–Meier survival curves of patients with PBL treated with RCHOP and CHOP categorized by IPI scores. A, Kaplan–Meier survival curves for OS of patients treated with RCHOP. B, Kaplan–Meier survival curves for PFS of patients treated with RCHOP. C, Kaplan–Meier survival curves for OS of patients treated with CHOP. D, Kaplan–Meier survival curves for PFS of patients treated with CHOP.

**Additional file 5: Figure S3.** Literature screening flow chart.

## Data Availability

The datasets generated during and/or analyzed during the current study are available from the corresponding author on reasonable request.

## Abbreviations

PBL: Primary breast lymphoma
PB- DLBCL: Primary breast diffuse large B-cell lymphoma
CNS: Central nervous system
R-CHOP: Rituximab-CHOP
IPI: International Prognostic Index
CT: Computed tomography
PET: Positron emission tomography
LDH: Lactate dehydrogenase
Bcl: B-cell lymphoma
CD: Cluster of differentiation
IHC: Immunohistochemistry assay
PFS: Progression-free survival
OS: Overall survival
MZL/MALT: Marginal zone lymphomas/mucosa-associated lymphoid tissue
FL: Follicular lymphoma
MCL: Mantle cell lymphoma
CR: Complete regression
PR: Partial response
SD: Stable disease
PD: Progression disease

## References

[1] Cheah CY, Campbell BA, Seymour JF (2014). Primary breast lymphoma. Cancer Treat Rev.

[2] Aviv A, Tadmor T, Polliack A (2013). Primary diffuse large B-cell lymphoma of the breast: looking at pathogenesis, clinical issues and therapeutic options. Ann Oncol.

[3] Domchek SM, Hecht JL, Fleming MD, Pinkus GS, Canellos GP (2002). Lymphomas of the breast: primary and secondary involvement. Cancer.

[4] Thomas A, Link BK, Altekruse S, Romitti PA, Schroeder MC (2017). Primary breast lymphoma in the United States: 1975–2013. J Natl Cancer Inst.

[5] Raj SD, Shurafa M, Shah Z, Raj KM, Fishman MDC, Dialani VM (2019). Primary and secondary breast lymphoma: clinical, pathologic, and multimodality imaging review. Radiographics.

[6] Ryan G, Martinelli G, Kuper-Hommel M, Tsang R, Pruneri G, Yuen K, Roos D, Lennard A, Devizzi L, Crabb S (2008). Primary diffuse large B-cell lymphoma of the breast: prognostic factors and outcomes of a study by the international extranodal lymphoma study group. Ann Oncol.

[7] Tomita N, Yokoyama M, Yamamoto W, Watanabe R, Shimazu Y, Masaki Y, Tsunoda S, Hashimoto C, Murayama K, Yano T (2012). Central nervous system event in patients with diffuse large B-cell lymphoma in the rituximab era. Cancer Sci.

[8] Hosein PJ, Maragulia JC, Salzberg MP, Press OW, Habermann TM, Vose JM, Bast M, Advani RH, Tibshirani R, Evens AM (2014). A multicentre study of primary breast diffuse large B-cell lymphoma in the rituximab era. Br J Haematol.

[9] Hu S, Song Y, Sun X, Su L, Zhang W, Jia J, Bai O, Yang S, Liang R, Li X (2018). Primary breast diffuse large B-cell lymphoma in the rituximab era: therapeutic strategies and patterns of failure. Cancer Sci.

[10] Hans CP, Weisenburger DD, Greiner TC, Gascoyne RD, Delabie J, Ott G, Müller-Hermelink HK, Campo E, Braziel RM, Jaffe ES (2004). Confirmation of the molecular classification of diffuse large B-cell lymphoma by immunohistochemistry using a tissue microarray. Blood.

[11] Sehn LH, Berry B, Chhanabhai M, Fitzgerald C, Gill K, Hoskins P, Klasa R, Savage KJ, Shenkier T, Sutherland J (2007). The revised international prognostic index (R-IPI) is a better predictor of outcome than the standard IPI for patients with diffuse large B-cell lymphoma treated with R-CHOP. Blood.

[12] Zhou Z, Sehn LH, Rademaker AW, Gordon LI, Lacasce AS, Crosby-Thompson A, Vanderplas A, Zelenetz AD, Abel GA, Rodriguez MA (2014). An enhanced international prognostic index (NCCN-IPI) for patients with diffuse large B-cell lymphoma treated in the rituximab era. Blood.

[13] Lin YC, Tsai CH, Wu JS, Huang CS, Kuo SH, Lin CW, Cheng AL (2009). Clinicopathologic features and treatment outcome of non-Hodgkin lymphoma of the breast–a review of 42 primary and secondary cases in Taiwanese patients. Leuk Lymphoma.

[14] Seker M, Bilici A, Ustaalioglu BO, Yilmaz B, Ozturk B, Ünal A, Dane F, Ozdemir NY, Elkiran ET, Kalender ME (2011). Clinicopathologic features of the nine patients with primary diffuse large B cell lymphoma of the breast. Arch Gynecol Obstet.

[15] Shao YB, Sun XF, He YN, Liu CJ, Liu H (2015). Clinicopathological features of thirty patients with primary breast lymphoma and review of the literature. Med Oncol.

[16] Luo H, Yi P, Wang W, Li K, Meng L, Li J, Zeng W, Tang M (2019). Clinicopathological features, treatment, and prognosis in primary diffuse large B cell lymphoma of the breast: a retrospective study of 46 patients. Med Sci Monit.

[17] Troppan KT, Schlick K, Deutsch A, Melchardt T, Egle A, Stojakovic T, Beham-Schmid C, Weiss L, Neureiter D, Wenzl K (2014). C-reactive protein level is a prognostic indicator for survival and improves the predictive ability of the R-IPI score in diffuse large B-cell lymphoma patients. Br J Cancer.

[18] Prochazka KT, Melchardt T, Posch F, Schlick K, Deutsch A, Beham-Schmid C, Weiss L, Gary T, Neureiter D, Klieser E (2016). NCCN-IPI score-independent prognostic potential of pretreatment uric acid levels for clinical outcome of diffuse large B-cell lymphoma patients. Br J Cancer.

[19] Melchardt T, Troppan K, Weiss L, Hufnagl C, Neureiter D, Tränkenschuh W, Hopfinger G, Magnes T, Deutsch A, Neumeister P (2015). A modified scoring of the NCCN-IPI is more accurate in the elderly and is improved by albumin and β2 -microglobulin. Br J Haematol.

[20] Troppan KT, Melchardt T, Deutsch A, Schlick K, Stojakovic T, Bullock MD, Reitz D, Beham-Schmid C, Weiss L, Neureiter D (2015). The significance of pretreatment anemia in the era of R-IPI and NCCN-IPI prognostic risk assessment tools: a dual-center study in diffuse large B-cell lymphoma patients. Eur J Haematol.

[21] Melchardt T, Troppan K, Weiss L, Hufnagl C, Neureiter D, Tränkenschuh W, Schlick K, Huemer F, Deutsch A, Neumeister P (2015). Independent prognostic value of serum markers in diffuse large B-cell lymphoma in the era of the NCCN-IPI. J Natl Compr Canc Netw.

[22] Harris NL, Jaffe ES, Diebold J, Flandrin G, Muller-Hermelink HK, Vardiman J, Lister TA, Bloomfield CD (1999). World Health Organization classification of neoplastic diseases of the hematopoietic and lymphoid tissues: report of the clinical advisory committee meeting-airlie house, virginia, November 1997. J Clin Oncol.

[23] Jia Y, Sun C, Liu Z, Wang W, Zhou X (2018). Primary breast diffuse large B-cell lymphoma: a population-based study from 1975 to 2014. Oncotarget.

[24] Sun Y, Joks M, Xu LM, Chen XL, Qian D, You JQ, Yuan ZY (2016). Diffuse large B-cell lymphoma of the breast: prognostic factors and treatment outcomes. Onco Targets Ther.

[25] Genco IS, Gur H, Hajiyeva S (2021). Lymphoma of the breast: a clinicopathologic analysis of 51 cases with a specific emphasis on patients with a history of breast carcinoma. Breast J.

[26] Martinelli G, Ryan G, Seymour JF, Nassi L, Steffanoni S, Alietti A, Calabrese L, Pruneri G, Santoro L, Kuper-Hommel M (2009). Primary follicular and marginal-zone lymphoma of the breast: clinical features, prognostic factors and outcome: a study by the international extranodal lymphoma study group. Ann Oncol.

[27] Valente I, Cantergiani F, Rinaldi A, Russo F, Mancini C, D'Aloia C (2020). Primitive marginal lymphoma of the breast. Breast J.

[28] Ganjoo K, Advani R, Mariappan MR, McMillan A, Horning S (2007). Non-Hodgkin lymphoma of the breast. Cancer.

[29] Miyoshi I, Yamamoto K, Saito T, Taguchi H (2006). Burkitt lymphoma of the breast. Am J Hematol.

[30] Elgaafary S, López C, Nagel I, Vater I, Bens S, Szczepanowski M, Aukema SM, Wagener R, Hopp L, Binder H (2021). Molecular characterization of Burkitt lymphoma in the breast or ovary. Leuk Lymphoma.

[31] Avilés A, Castañeda C, Neri N, Cleto S, Nambo MJ (2007). Rituximab and dose dense chemotherapy in primary breast lymphoma. Haematologica.

[32] Liu PP, Wang KF, Jin JT, Bi XW, Sun P, Wang Y, Yang H, Li ZM, Jiang WQ, Xia Y (2018). Role of radiation therapy in primary breast diffuse large B-cell lymphoma in the Rituximab era: a SEER database analysis. Cancer Med.

[33] Lin Y, Guo XM, Shen KW, Wang JL, Jiang GL (2006). Primary breast lymphoma: long-term treatment outcome and prognosis. Leuk Lymphoma.

[34] Zhang T, Zhang Y, Fei H, Shi X, Wang L, Wang P, Yu J, Shen Y, Feng S (2021). Primary breast double-hit lymphoma management and outcomes: a real-world multicentre experience. Cancer Cell Int.

[35] Yhim HY, Kim JS, Kang HJ, Kim SJ, Kim WS, Choi CW, Eom HS, Kim JA, Lee JH, Won JH (2012). Matched-pair analysis comparing the outcomes of primary breast and nodal diffuse large B-cell lymphoma in patients treated with rituximab plus chemotherapy. Int J Cancer.

[36] Zhao P, Zhu L, Song Z, Wang X, Ma W, Zhu X, Qiu L, Li L, Zhou S, Qian Z (2020). Combination of baseline total metabolic tumor volume measured on FDG-PET/CT and β2-microglobulin have a robust predictive value in patients with primary breast lymphoma. Hematol Oncol.

[37] Ou CW, Shih LY, Wang PN, Chang H, Kuo MC, Tang TC, Wu JH, Lin TL, Hung YS, Dunn P (2014). Primary breast lymphoma: a single-institute experience in Taiwan. Biomed J.

[38] Zhang N, Cao C, Zhu Y, Liu P, Liu L, Lu K, Luo J, Zhou N (2016). Primary breast diffuse large B-cell lymphoma in the era of rituximab. Onco Targets Ther.

