# Supplementary material for: The prognostic value of IPI in patients with primary breast lymphoma, a multicenter retrospective study
Source: Cancer Cell Int. 2022 Nov 15;22:357. doi: 10.1186/s12935-022-02772-y (PMC9664603; doi:10.1186/s12935-022-02772-y)
Supplement: Supplementary file 2 — Additional file 2: Table S2. Basic information of studies included in the literature review. [file 12935_2022_2772_MOESM2_ESM.docx]

Table 2. Basic information of studies included in the literature review.

| Author, year | N | FU (Year) | Age | | | IPI | | B symptoms | | 5-year OS | 5-year PFS | Treatment (N) | | | |
| --- | --- | --- | --- | --- | --- | --- | --- | --- | --- | --- | --- | --- | --- | --- | --- |
|  |  |  | < 60 | | ≥ 60 | 0-1 | 2-5 | Absent | Present |  |  | Surgery | R+ChT | ChT | RT |
| Ryan, 2008[1] | 204 | 5.5 | 81 | | 123 | 129 | 37 | 195 | 9 | 46% | 36% | 9 | 0 | 143 | 130 |
| Ludmir, 2019[2] | 11 | 8 | 62 (42-75) | | | 5 | 6 | 11 | 0 | NA | NA | 1 | NA | NA | 8 |
| Hu, 2018[3] | 108 | 3.2 | 47 (16-85) | | | 78 | 13 | 103 | 5 | 77.3% | 61.2% | 21 | 66 | 108 | 39 |
| Zhang, 2021[4] | 36 | 2.8 | 18 | | 11 | 3 | 15 | 14 | 15 | NA | NA | 0 | 8 | 8 | 14 |
| Luo, 2019[5] | 46 | 3.4 | 28 | | 18 | 33 | 13 | 44 | 2 | 36.2% | 29.1% | 38 | 16 | 46 | 12 |
| Ludmir, 2018[2] | 25 | 4.5 | 55 (26–83) | | | 16 | 6 | 24 | 1 | 71.9% | 42.4$ | 5 | 16 | 24 | 13 |
| Niitsu, 2008[6] | 30 | 5.5 | 57 (24–77) | | | 23 | 7 | 27 | 3 | 87.0% | 77.0% | NA | 11 | 30 | 18 |
| Yhim, 2020*[7] | 33 | 3.8 | 50(29-75) | | | 28 | 5 | 31 | 2 | 93.5% | 81.3% | NA | 32 | 32 | NA |
| Zhang, 2016[8] | 24 | 5.0 | 50 (24-69) | | | 19 | 5 | 23 | 1 | 78.9% | 79.2% | 17 | 10 | 23 | 12 |
| Hosein, 2014[9] | 76 | 4.5 | 62 (17-87) | | | 54 | 22 | 74 | 2 | 75.0% | 66.0% | NA | 47 | 65 | 48 |
| Ou, 2015[10] | 23 | 3.8 | 16 | | 5 | 21 | 2 | 22 | 1 | 57.1% | 57.1% | 3 | 7 | 19 | 1 |
| Zhao, 2020[11] | 64 | 5 | 51 | | 13 | 54 | 5 | 53 | 11 | 73.4% | 62.5% | 29 | 39 | 59 | 39 |
| Zhang, 2017[12] | 29 | 5.5 | 50 (24-69) | | | 21 | 8 | 27 | 2 | 78.1% | 78.4% | 21 | 11 | 27 | 13 |
| Shao, 2015[13] | 30 | 2.7 | 45 (18-74) | | | 17 | 13 | 25 | 5 | 48.0% | 32.0% | NA | 13 | 24 | 5 |
| Avilés, 2007*[14] | 32 | 5.4 | 16 | | 16 | 32 | 0 | NA | NA | 63.0% | 75.0% | NA | 0 | 32 | NA |
| Aviles, 2012[15] | 104 | NA | NA | | NA | 104 | 0 | NA | NA | 52.0% | 66.0% | NA | 49 | 55 | 0 |
| Martinelli, 2009[16] | 60 | 4 | 25 | | 35 | 36 | NA | 56 | 4 | 92.0% | 56.0% | 40 | NA | 25 | 36 |
| Yhim, 2012[17] | 26 | 2.9 | 56 (21-79) | | | 20 | 5 | 24 | 1 | 82.2% | 70.0% | NA | 6 | 25 | 10 |
| Liu, 2020[18] | 370 | 5.7 | 114 | 256 | | NA | NA | NA | NA | 81.2% | 95.4% | 71 | NA | 63 | 142 |
| Lalani, 2018[19] | 35 | 5.8 | 66 (35-86) | | | NA | NA | 31 | 4 | 70.0% | NA | 15 | 10 | 15 | 30 |
| Franco, 2016[20] | 55 | 4.7 | 28 | 25 | | NA | NA | 51 | 2 | 76.0% | 73.0% | 14 | 39 | 35 | 20 |
| Radkan, 2014[21] | 28 | NA | 67.5 (35-95) | | | NA | NA | NA | NA | 82.0% | 75.0% | 17 | NA | 15 | 16 |
| Jeanneret-Sozzi, 2008[22] | 84 | 4.7 | 42 | 12 | | NA | NA | NA | NA | 53.0% | 59.0% | 21 | 0 | 59 | 51 |
| Lin, 2006[23] | 32 | 6.3 | 43 (22-76) | | | NA | NA | NA | NA | 63.9% | 58.6% | 32 | NA | 28 | 20 |
| Jennings, 2007[24] | 465 | 4.0 | 54 (17-95) | | | NA | NA | NA | NA | NA | 44.5% | 156 | 0 | 323 | 218 |

*Prospective studies

FU, follow-up; IPI, International Prognostic Index; PFS, progression-free survival; OS, overall survival; ChT, chemotherapy; R, rituximab; NA, not available

Reference

1. Ryan G, Martinelli G, Kuper-Hommel M, Tsang R, Pruneri G, Yuen K, Roos D, Lennard A, Devizzi L, Crabb S *et al*: **Primary diffuse large B-cell lymphoma of the breast: prognostic factors and outcomes of a study by the International Extranodal Lymphoma Study Group**. *Ann Oncol* 2008, **19**(2):233-241.

2. Ludmir EB, Milgrom SA, Pinnix CC, Gunther JR, Westin J, Fayad LE, Khoury JD, Medeiros LJ, Dabaja BS, Nastoupil LJ: **Emerging Treatment Strategies for Primary Breast Extranodal Marginal Zone Lymphoma of Mucosa-associated Lymphoid Tissue**. *Clin Lymphoma Myeloma Leuk* 2019, **19**(4):244-250.

3. Hu S, Song Y, Sun X, Su L, Zhang W, Jia J, Bai O, Yang S, Liang R, Li X *et al*: **Primary breast diffuse large B-cell lymphoma in the rituximab era: Therapeutic strategies and patterns of failure**. *Cancer Sci* 2018, **109**(12):3943-3952.

4. Zhang T, Zhang Y, Fei H, Shi X, Wang L, Wang P, Yu J, Shen Y, Feng S: **Primary breast double-hit lymphoma management and outcomes: a real-world multicentre experience**. *Cancer Cell Int* 2021, **21**(1):498.

5. Luo H, Yi P, Wang W, Li K, Meng L, Li J, Zeng W, Tang M: **Clinicopathological Features, Treatment, and Prognosis in Primary Diffuse Large B Cell Lymphoma of the Breast: A Retrospective Study of 46 Patients**. *Med Sci Monit* 2019, **25**:8671-8682.

6. Niitsu N, Okamoto M, Nakamine H, Hirano M: **Clinicopathologic features and treatment outcome of primary breast diffuse large B-cell lymphoma**. *Leuk Res* 2008, **32**(12):1837-1841.

7. Yhim HY, Yoon DH, Kim SJ, Yang DH, Eom HS, Kim KH, Park Y, Kim JS, Kim HJ, Suh C *et al*: **First-Line Treatment for Primary Breast Diffuse Large B-Cell Lymphoma Using Immunochemotherapy and Central Nervous System Prophylaxis: A Multicenter Phase 2 Trial**. *Cancers (Basel)* 2020, **12**(8).

8. Zhang N, Cao C, Zhu Y, Liu P, Liu L, Lu K, Luo J, Zhou N: **Primary breast diffuse large B-cell lymphoma in the era of rituximab**. *Onco Targets Ther* 2016, **9**:6093-6097.

9. Hosein PJ, Maragulia JC, Salzberg MP, Press OW, Habermann TM, Vose JM, Bast M, Advani RH, Tibshirani R, Evens AM *et al*: **A multicentre study of primary breast diffuse large B-cell lymphoma in the rituximab era**. *Br J Haematol* 2014, **165**(3):358-363.

10. Ou CW, Shih LY, Wang PN, Chang H, Kuo MC, Tang TC, Wu JH, Lin TL, Hung YS, Dunn P: **Primary breast lymphoma: a single-institute experience in Taiwan**. *Biomed J* 2014, **37**(5):321-325.

11. Zhao P, Zhu L, Song Z, Wang X, Ma W, Zhu X, Qiu L, Li L, Zhou S, Qian Z *et al*: **Combination of baseline total metabolic tumor volume measured on FDG-PET/CT and β2-microglobulin have a robust predictive value in patients with primary breast lymphoma**. *Hematol Oncol* 2020, **38**(4):493-500.

12. Zhang N, Cao C, Zhu Y, Liu P, Liu L, Lu K, Luo J, Zhou N: **Primary breast lymphoma: A single center study**. *Oncol Lett* 2017, **13**(2):1014-1018.

13. Shao YB, Sun XF, He YN, Liu CJ, Liu H: **Clinicopathological features of thirty patients with primary breast lymphoma and review of the literature**. *Med Oncol* 2015, **32**(2):448.

14. Avilés A, Castañeda C, Neri N, Cleto S, Nambo MJ: **Rituximab and dose dense chemotherapy in primary breast lymphoma**. *Haematologica* 2007, **92**(8):1147-1148.

15. Aviles A, Neri N, Nambo MJ: **The role of genotype in 104 cases of diffuse large B-cell lymphoma primary of breast**. *Am J Clin Oncol* 2012, **35**(2):126-129.

16. Martinelli G, Ryan G, Seymour JF, Nassi L, Steffanoni S, Alietti A, Calabrese L, Pruneri G, Santoro L, Kuper-Hommel M *et al*: **Primary follicular and marginal-zone lymphoma of the breast: clinical features, prognostic factors and outcome: a study by the International Extranodal Lymphoma Study Group**. *Ann Oncol* 2009, **20**(12):1993-1999.

17. Yhim HY, Kim JS, Kang HJ, Kim SJ, Kim WS, Choi CW, Eom HS, Kim JA, Lee JH, Won JH *et al*: **Matched-pair analysis comparing the outcomes of primary breast and nodal diffuse large B-cell lymphoma in patients treated with rituximab plus chemotherapy**. *Int J Cancer* 2012, **131**(1):235-243.

18. Liu H, Zhang J, Quan L, Cao L, Miao Y, Zhao X, Shen H, Wang L, Xu W, Li J *et al*: **Conventional Treatments Cannot Improve Outcomes of Early-Stage Primary Breast Marginal Zone Lymphoma**. *Front Oncol* 2020, **10**:609512.

19. Lalani N, Winkfield KM, Soto DE, Yeap BY, Ng AK, Mauch PM, Jimenez RB: **Management and outcomes of women diagnosed with primary breast lymphoma: a multi-institution experience**. *Breast Cancer Res Treat* 2018, **169**(1):197-202.

20. Franco Pérez F, Lavernia J, Aguiar-Bujanda D, Miramón J, Gumá J, Álvarez R, Gómez-Codina J, Arroyo FG, Llanos M, Marin M *et al*: **Primary Breast Lymphoma: Analysis of 55 Cases of the Spanish Lymphoma Oncology Group**. *Clin Lymphoma Myeloma Leuk* 2017, **17**(3):186-191.

21. Radkani P, Joshi D, Paramo JC, Mesko TW: **Primary breast lymphoma: 30 years of experience with diagnosis and treatment at a single medical center**. *JAMA Surg* 2014, **149**(1):91-93.

22. Jeanneret-Sozzi W, Taghian A, Epelbaum R, Poortmans P, Zwahlen D, Amsler B, Villette S, Belkacémi Y, Nguyen T, Scalliet P *et al*: **Primary breast lymphoma: patient profile, outcome and prognostic factors. A multicentre Rare Cancer Network study**. *BMC Cancer* 2008, **8**:86.

23. Lin Y, Guo XM, Shen KW, Wang JL, Jiang GL: **Primary breast lymphoma: long-term treatment outcome and prognosis**. *Leuk Lymphoma* 2006, **47**(10):2102-2109.

24. Jennings WC, Baker RS, Murray SS, Howard CA, Parker DE, Peabody LF, Vice HM, Sheehan WW, Broughan TA: **Primary breast lymphoma: the role of mastectomy and the importance of lymph node status**. *Ann Surg* 2007, **245**(5):784-789.
