# Supplementary figures and images for: The prognostic value of IPI in patients with primary breast lymphoma, a multicenter retrospective study
Source: Cancer Cell Int. 2022 Nov 15;22:357. doi: 10.1186/s12935-022-02772-y (PMC9664603; doi:10.1186/s12935-022-02772-y)

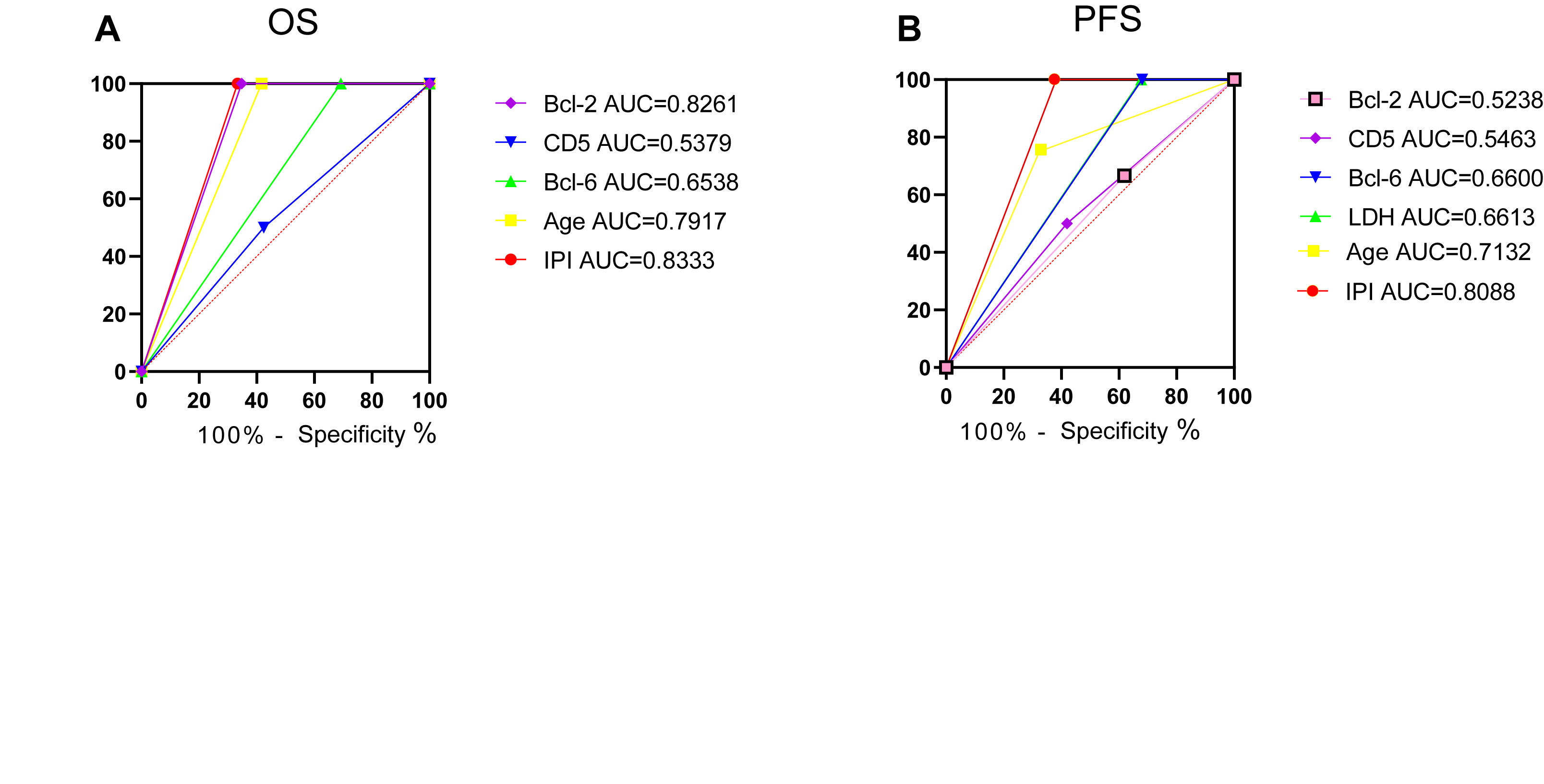

Supplement: Supplementary file 3 — Additional file 3: Figure S1. ROC curves for 5-year survival of IPI and predictive factors significant in univariate analysis. A, ROC curves for 5-year OS of IPI and predictive factors significant in univariate analysis. B, ROC curves for 5-year PFS of IPI and predictive factors significant in univariate analysis. [file 12935_2022_2772_MOESM3_ESM.tif]

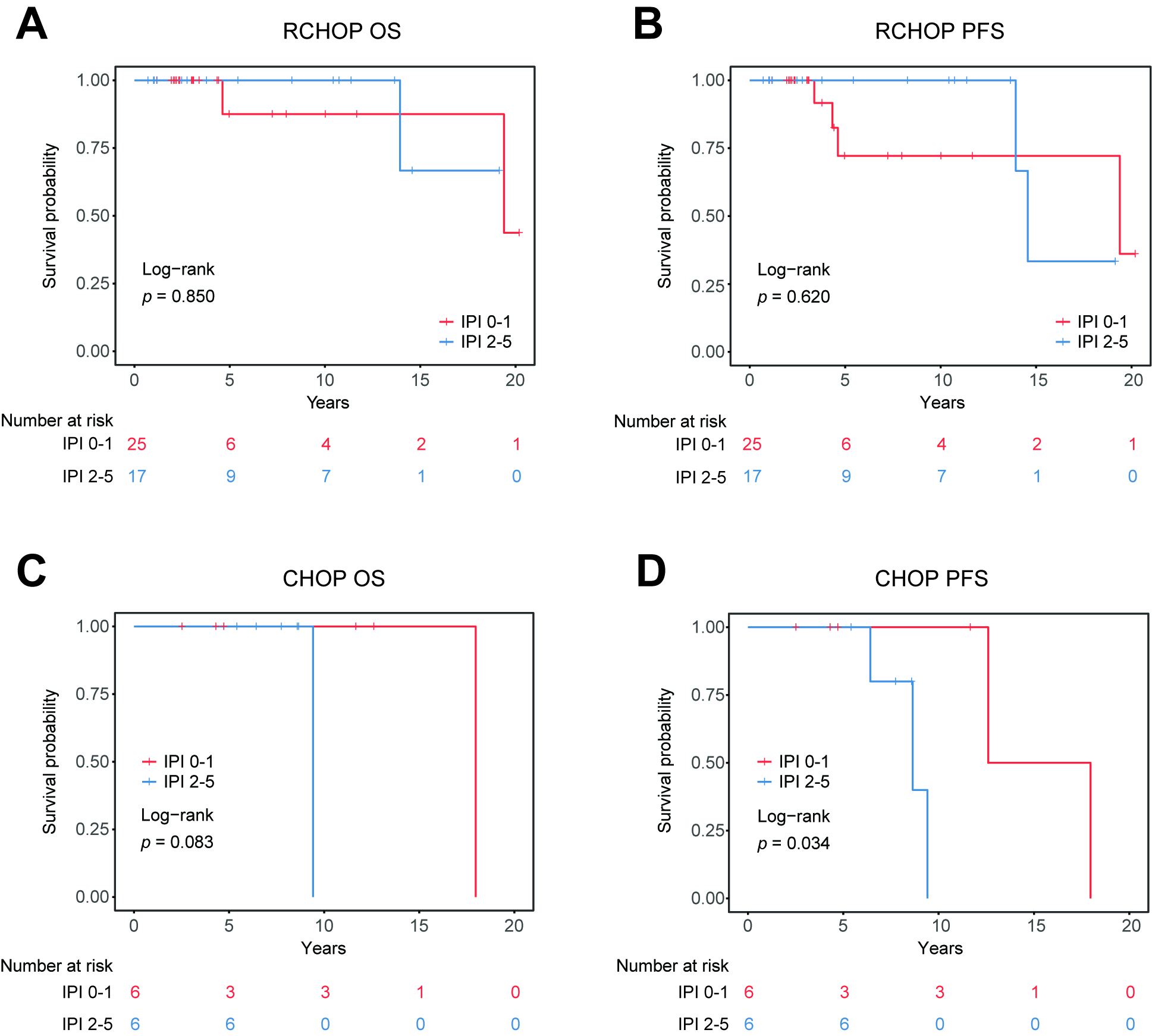

Supplement: Supplementary file 4 — Additional file 4: Figure S2. Kaplan–Meier survival curves of patients with PBL treated with RCHOP and CHOP categorized by IPI scores. A, Kaplan–Meier survival curves for OS of patients treated with RCHOP. B, Kaplan–Meier survival curves for PFS of patients treated with RCHOP. C, Kaplan–Meier survival curves for OS of patients treated with CHOP. D, Kaplan–Meier survival curves for PFS of patients treated with CHOP. [file 12935_2022_2772_MOESM4_ESM.tif]

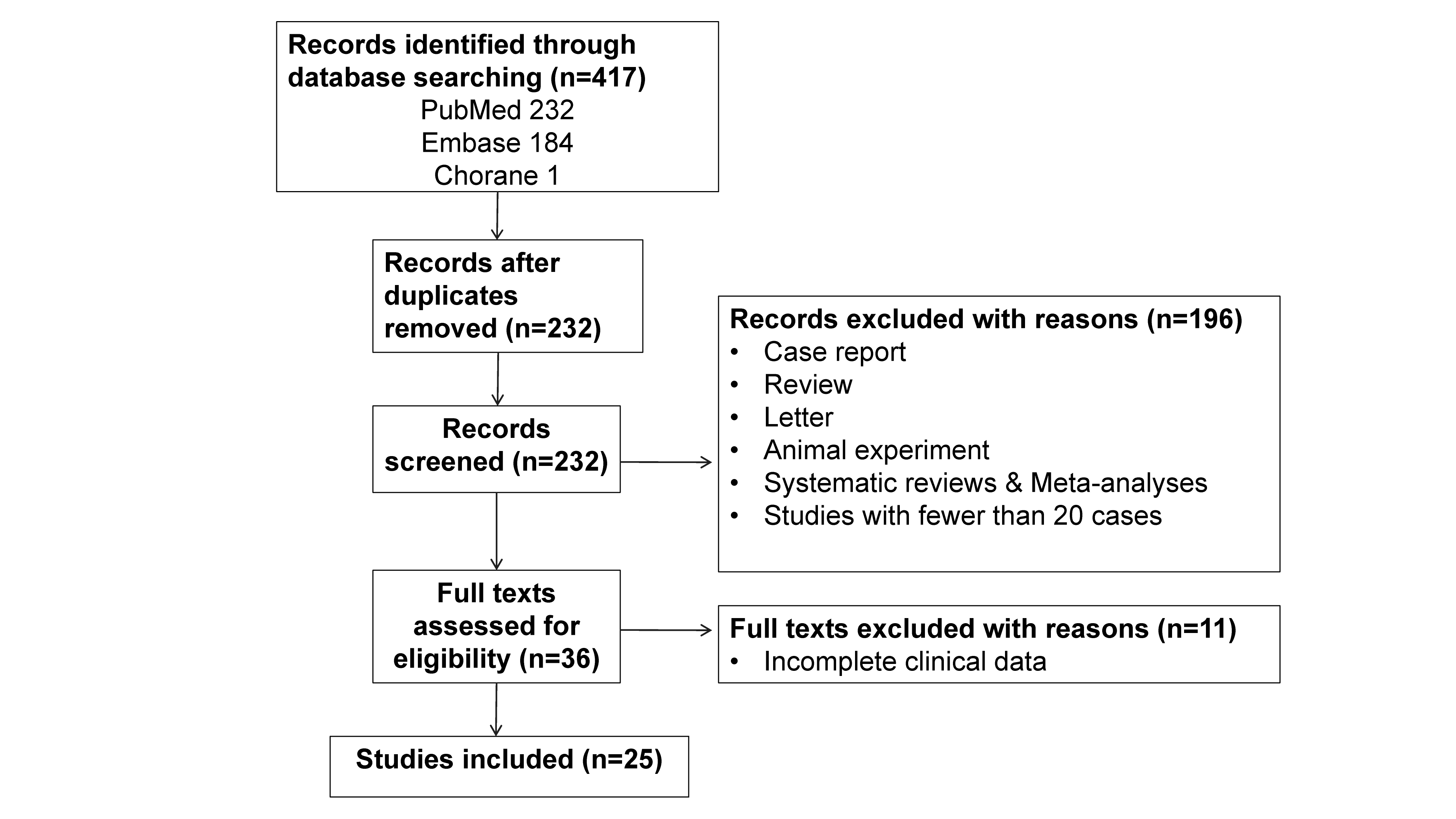

Supplement: Supplementary file 5 — Additional file 5: Figure S3. Literature screening flow chart. [file 12935_2022_2772_MOESM5_ESM.tif]
